# Influence of biological sex on neuroinflammatory dynamics in the aging brain

**DOI:** 10.3389/fnagi.2025.1670175

**Published:** 2025-08-29

**Authors:** Ludmila Müller, Svetlana Di Benedetto, Viktor Müller

**Affiliations:** Center for Lifespan Psychology, Max Planck Institute for Human Development, Berlin, Germany

**Keywords:** aging brain, neuroinflammation, immunosenescence, glial cells, neurons, immune cells, cytokines, neurodegenerative diseases

## Abstract

The aging brain undergoes complex neuroinflammatory changes that are increasingly recognized as contributing factors to the development and progression of neurodegenerative diseases. Emerging research reveals that biological sex profoundly shapes these neuroinflammatory dynamics, resulting in distinct trajectories of immune function, glial activity, and neural vulnerability in males and females. This mini-review focuses on recent advances in understanding the interplay of hormonal, genetic, and epigenetic factors that drive sex-specific differences of neuroinflammatory processes in aging brain. We begin by describing the hallmarks of neuroinflammation, including chronic activation of glial cells and the loss of inflammatory resolution. We provide a brief overview of age-related changes in microglial and astrocyte function, along with systemic influences such as immunosenescence, inflammaging, dysbiosis, and increased blood–brain barrier permeability. Building on this foundation, we examine sex-dependent differences in immune aging, CNS immune surveillance, and hormonal regulation of glial activity, particularly in the context of menopause and andropause. Particular attention is given to how these mechanisms drive sex-specific differences in the pathophysiology of neuroinflammation—a key contributor to many neurodegenerative diseases. Finally, we address key methodological challenges—such as the underrepresentation of females in preclinical models and limited sex-stratified clinical analyses—that constrain our understanding of sex-specific neuroinflammation in aging. By integrating sex as a critical biological variable and exploring systems-based approaches such as multilayer network models, this review highlights the importance of sex-informed research to better understand, prevent, and treat neuroinflammatory and neurodegenerative conditions in aging populations.

## 1 Introduction

Aging is accompanied by wide-ranging physiological changes, among which chronic, low-grade inflammation—often referred to as “inflammaging”—has emerged as a key contributor to the onset and progression of age-related and neurodegenerative diseases (Franceschi and Campisi, 2014; Franceschi et al., 2018). Within the central nervous system (CNS), this aging-associated inflammatory milieu manifests as neuroinflammation, characterized by glial cell reactivity, altered cytokine profiles, and compromised neuronal-glial communication (Müller and Di Benedetto, 2021; Müller and Di Benedetto, 2024; Murdaca et al., 2022). While much attention has been given to the cellular and molecular mechanisms underlying neuroinflammation, there is increasing recognition that biological sex significantly modulates these processes, leading to distinct trajectories of neuroimmune aging in males and females (Di Benedetto et al., 2019; Han et al., 2021; Hanamsagar and Bilbo, 2016).

Sex differences in brain structure, immune function, and hormonal signaling emerge from a multifaceted interplay among genetic, epigenetic, endocrine, and environmental influences (Kundakovic and Tickerhoof, 2024; Bhattacharya et al., 2024; Marrocco et al., 2020; McCarthy et al., 2017; Calabro et al., 2023; Ruigrok et al., 2014). These differences extend into aging and profoundly influence how the aging brain responds to pathological stressors (Hanamsagar and Bilbo, 2016; Huang et al., 2024). For instance, Alzheimer’s disease (AD) disproportionately affects women in both its prevalence and rate of cognitive decline, whereas Parkinson’s disease (PD) is more common in men and often presents with differing symptom profiles; similarly, multiple sclerosis (MS) occurs more frequently in women, but tends to follow a more aggressive, neurodegenerative course in men (Laws et al., 2018; Cattaneo and Pagonabarraga, 2025; de Souza et al., 2022; Boccalini et al., 2025; Ribbons et al., 2015; Gilli et al., 2020). These disparities suggest that sex is not just a demographic variable but a fundamental biological factor influencing the onset, progression, and outcomes of neuroinflammatory processes.

Recent evidence points to sex-specific mechanisms driving neuroimmune aging, including divergent states of microglial and astrocyte activation, sex-hormone-dependent cytokine signaling, and distinct glial gene expression profiles (Han et al., 2021; Fritz Garcia et al., 2024; Bonkhoff et al., 2025). Examples include the finding that estrogens can directly influence microglial inflammatory responses, revealing potential hormonal mechanisms for sex-modulated neuroinflammation (Fritz Garcia et al., 2024). However, despite the growing body of evidence, most preclinical studies have long overlooked sex as a critical biological variable, leading to the underrepresentation of females in preclinical and clinical neuroscience studies. This has led to an incomplete understanding of how sex-dependent neuroinflammation influences aging and disease (Fritz Garcia et al., 2024; Sosinsky et al., 2022), resulting in a significant knowledge gap that may hinder the development of effective, personalized therapeutic strategies.

This mini-review aims to highlight recent findings on sex-specific modulation of neuroinflammation during aging, with a focus on the cellular and molecular mechanisms underlying these differences. We will explore how genetic, epigenetic, and hormonal influences shape immune and CNS responses in aging males and females. Particular attention is given to how these mechanisms drive sex-specific differences in the pathophysiology of neuroinflammation—a key contributor to many neurodegenerative diseases—underscoring the urgent need for sex-informed approaches in both research and clinical practice. Recognizing and integrating these differences is essential not only for understanding disease vulnerability and progression across sexes but also for optimizing diagnostic strategies and developing targeted, more effective therapeutic interventions.

## 2 Neuroinflammation and aging: an overview

**Figure 1:**
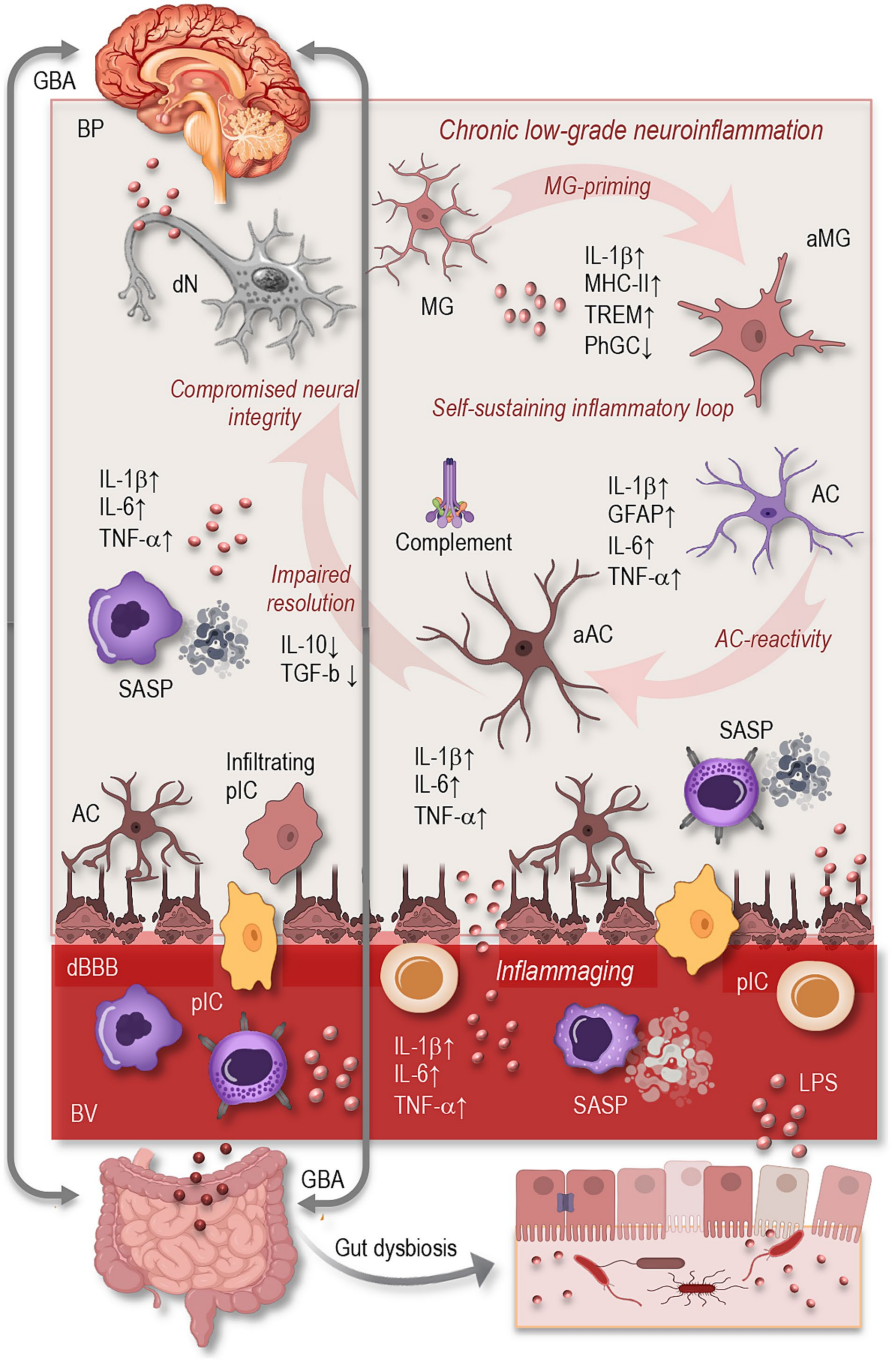
Overview of neuroinflammation in the aging brain. Aging induces profound alterations in central and systemic immune regulation, leading to a state of chronic, low-grade neuroinflammation. Key features include microglial activation (↑MHC-II, IL-1β, TREM2; ↓phagocytosis), astrocytic reactivity (↑GFAP, cytokines, complement), and impaired resolution of inflammation. These glial changes form a self-sustaining inflammatory loop that compromises neuronal integrity and cognitive resilience. Systemically, inflammaging (↑IL-6, TNF-α, CRP) and immune senescence (SASP phenotype in monocytes/T cells) amplify CNS inflammation, especially as BBB permeability increases. Peripheral drivers such as gut dysbiosis and microbial endotoxins (e.g., LPS) further exacerbate neuroinflammation via the gut–brain axis. Together, these mechanisms contribute to synaptic loss, impaired neurogenesis, and increased vulnerability to neurodegeneration. GBA, gut-brain axis; BP, brain parenchyma; dN, degenerating neuron; MG, microglia; aMG, activated microglia; AC, astrocyte; aAC, activated astrocyte; dN, degenerating neuron; IL, interleukin; MHC, major histocompatibility complex; TREM, triggering receptor expressed on myeloid cells; PhGC, phagocytose; TNF, tumor necrosis factor; TGF, tumor growth factor; cBBB, compromised blood–brain-barrier; SASP, senescence-associated secretory phonotype; GFAP, glial fibrillary acidic protein; pIC, peripheral immune cells; BV, blood vessel; LPS, lipopolysaccharides.

Aging is a dynamic and multifactorial process that profoundly affects the immune landscape of the CNS. A defining feature of the aging brain is the gradual emergence of chronic, low-grade inflammation—commonly referred to as neuroinflammation—which has been increasingly recognized as both a hallmark of aging and a central driver of neurodegenerative processes. Unlike the acute and tightly regulated inflammatory responses that support tissue repair following injury or infection, neuroinflammation in aging is prolonged and poorly controlled. It arises from intrinsic changes in the brain’s immune architecture, particularly involving glial cells, and is further sustained by systemic inflammatory signals (Figure 1). These include chronic low-grade inflammation associated with immunosenescence, increased blood–brain barrier permeability, and the influx of peripheral immune mediators. Together, these central and peripheral factors establish a self-reinforcing inflammatory loop that compromises neural homeostasis and increases susceptibility to cognitive decline and neurodegeneration (Di Benedetto et al., 2017; DiSabato et al., 2016; Colonna and Butovsky, 2017; Ransohoff, 2016; Teleanu et al., 2022; Wendimu and Hooks, 2022).

### 2.1 Hallmarks of neuroinflammation in the aging brain

In the aged brain, neuroinflammatory processes are characterized by a steady upregulation of pro-inflammatory mediators, including interleukin-1β (IL-1β), tumor necrosis factor-α (TNF-α), and interleukin-6 (IL-6), even in the absence of overt pathology. This elevation of baseline inflammation reflects not only increased cytokine production but also a decline in anti-inflammatory counter-regulation, such as diminished interleukin-10 (IL-10) and transforming growth factor-beta (TGF-β) signaling (Figure 1). Additionally, the aging brain exhibits a loss of homeostatic regulation in glial populations and impaired mechanisms for resolving inflammation or clearing cellular debris (Müller and Di Benedetto, 2024; Jurgens and Johnson, 2012; Mayne et al., 2020; Shafqat et al., 2023).

Crucially, neuroinflammation is not an isolated event but an early and persistent contributor to the progression of age-associated diseases (Shafqat et al., 2023; Heneka et al., 2024). Chronic activation of inflammatory pathways can disrupt cellular signaling, impair synaptic plasticity, reduce adult neurogenesis in regions such as the hippocampus, and compromise the structural and functional integrity of neuronal networks (Barrientos et al., 2015; Müller et al., 2025). These alterations weaken the brain’s capacity for adaptive remodeling and cognitive resilience, contributing to deficits in learning, memory, and executive function (Müller and Di Benedetto, 2024; Müller and Di Benedetto, 2025a; Abdelhamed et al., 2025; Dhapola et al., 2021; Pamies et al., 2021; Yirmiya and Goshen, 2011).

Importantly, such changes are not purely reactive but represent a maladaptive trajectory that unfolds gradually over time. With advancing age, glial cells become increasingly dysregulated, adopting pro-inflammatory phenotypes that perpetuate a hostile environment for neurons (Müller and Di Benedetto, 2024; Shafqat et al., 2023; Müller and Di Benedetto, 2025a; von Bernhardi et al., 2015). This chronic state of immune activation occurs even in the absence of evident neurodegenerative pathology and may act as a silent initiator of vulnerability, setting the stage for more pronounced disease processes when additional genetic, environmental, or metabolic stressors are introduced (Norden and Godbout, 2013). In this context, neuroinflammation should be viewed not only as a consequence but also as a critical precursor and amplifier of age-related cognitive and neurodegenerative disorders.

### 2.2 Glial cell dysregulation with age

Glial cells—particularly microglia and astrocytes—play a central role in the development and perpetuation of age-related neuroinflammation. These cells, once thought to function merely as support for neurons, are now understood to be highly dynamic participants in immune surveillance, synaptic remodeling, and metabolic regulation (Singh, 2022; Song and Dityatev, 2018). However, aging drives them into increasingly reactive and dysfunctional states (Figure 1).

Microglia, the brain’s resident macrophages, undergo a profound phenotypic transformation with age. They adopt a pro-inflammatory transcriptional profile, characterized by elevated expression of major histocompatibility complex class II (MHC-II), IL-1β, and triggering receptor expressed on myeloid cells 2 (TREM2). Morphologically, aged microglia display shorter, less motile processes and exhibit reduced capacity for phagocytosis and debris clearance (Singh, 2022; Rachmian et al., 2024; Ulrich and Holtzman, 2016). At the same time, their heightened sensitivity to secondary stimuli—often referred to as “priming”—makes them disproportionately responsive to minor insults, resulting in an exaggerated inflammatory response (Norden and Godbout, 2013; Norden et al., 2015; Perry and Holmes, 2014).

Astrocytes, too, exhibit significant changes in aging (Figure 1). While these cells are essential for maintaining extracellular homeostasis, regulating synaptic function, and providing metabolic support, their aging counterparts often shift toward a reactive phenotype. This is marked by increased expression of glial fibrillary acidic protein (GFAP), altered calcium signaling, and a loss of neuroprotective functions. Aged astrocytes also begin to secrete pro-inflammatory cytokines and complement proteins, contributing to microglial activation and synaptic pruning. Some astrocytes acquire a neurotoxic profile, which actively promotes neuronal damage and synaptic loss (DiSabato et al., 2016; Durkee and Araque, 2019; Hol and Pekny, 2015; Kolliker-Frers et al., 2021; Lee et al., 2023; Liddelow et al., 2017). The intercellular dialog between microglia and astrocytes, mediated by cytokines and chemokines, becomes increasingly dysregulated with age, forming a self-perpetuating cycle of glial reactivity and neurotoxicity.

### 2.3 Systemic influences on the aging brain

Beyond the CNS itself, systemic physiological changes in aging have a profound impact on brain immunity. One of the most influential processes is inflammaging, a term coined to describe the chronic, systemic elevation of inflammatory mediators such as IL-6, TNF-α, and C-reactive protein (CRP) in the elderly. This background inflammation not only reflects immune senescence but also exerts direct effects on the brain by enhancing glial reactivity and altering neural homeostasis (Franceschi and Campisi, 2014; Müller and Di Benedetto, 2024; Franceschi et al., 2018).

Another critical factor is the age-associated breakdown of the blood–brain barrier (BBB), a selective interface that normally protects the brain from peripheral insults. In aging, BBB integrity becomes compromised, permitting the influx of circulating cytokines, immune cells, and microbial components into the CNS (Figure 1). This permeability disrupts the brain’s immune privilege and facilitates the initiation or amplification of neuroinflammatory responses (Montagne et al., 2015).

Moreover, senescent peripheral immune cells, particularly monocytes and T lymphocytes, begin to exhibit a characteristic senescence-associated secretory phenotype (SASP), which contributes significantly to the inflammaging. This phenotype is marked by the sustained release of pro-inflammatory cytokines (e.g., IL-6, IL-1β, TNF-α), chemokines (e.g., CCL2, CXCL10), growth factors, and matrix-degrading enzymes such as metalloproteinases (Müller and Di Benedetto, 2021; Freund et al., 2010; Müller and Di Benedetto, 2023; Müller L. et al., 2019). These circulating factors do not remain confined to peripheral compartments but can cross BBB, especially as its integrity becomes increasingly compromised with age (Montagne et al., 2015). As a result, SASP factors can promote microglial priming, astrocyte reactivity, and endothelial dysfunction within the CNS, even in the absence of direct infection or injury (Di Benedetto et al., 2017; Alkhalifa et al., 2023; Huang et al., 2021; Knox et al., 2022).

Importantly, this systemic-to-central signaling cascade can accelerate neural aging by perpetuating a state of immune alertness in the brain, increasing vulnerability to cognitive decline and neurodegenerative disease. The accumulation of senescent immune cells in the periphery, therefore, acts as a remote but powerful modulator of brain health, reinforcing neuroinflammatory processes already active within the CNS. This underscores the growing recognition that age-related neuroinflammation is not solely a product of local glial dysregulation but is integrally shaped by peripheral immune aging and systemic inflammatory tone (Di Benedetto et al., 2017; Norden et al., 2015; Perry and Holmes, 2014; Franceschi et al., 2018; Di Benedetto et al., 2019).

Changes in the gut microbiota represent an increasingly recognized factor contributing to systemic and CNS inflammation during aging. As individuals grow older, the composition and diversity of the gut microbiome undergo significant alterations, often marked by a decline in beneficial commensal bacteria and an overrepresentation of pro-inflammatory species (Figure 1). This microbial imbalance—termed dysbiosis—is frequently accompanied by increased intestinal permeability, or “leaky gut,” which allows bacterial endotoxins such as lipopolysaccharides (LPS) to enter the circulation. These endotoxins act as potent immune activators, triggering systemic inflammation that can, in turn, influence the brain via the gut–brain axis (Bano et al., 2024; Chenghan et al., 2025; Müller and Di Benedetto, 2025b). Through both humoral and neural pathways, microbial metabolites and circulating pro-inflammatory signals can cross or modulate the BBB, promoting glial cell activation and amplifying neuroinflammatory responses. This bidirectional communication between the gut and brain suggests that age-related dysbiosis may act as a chronic peripheral driver of microglial priming and astrocytic reactivity, thereby compounding the neuroinflammatory burden characteristic of the aging brain (Freund et al., 2010; Müller and Di Benedetto, 2025b).

## 3 Sex differences in immune and CNS responses

**Figure 2:**
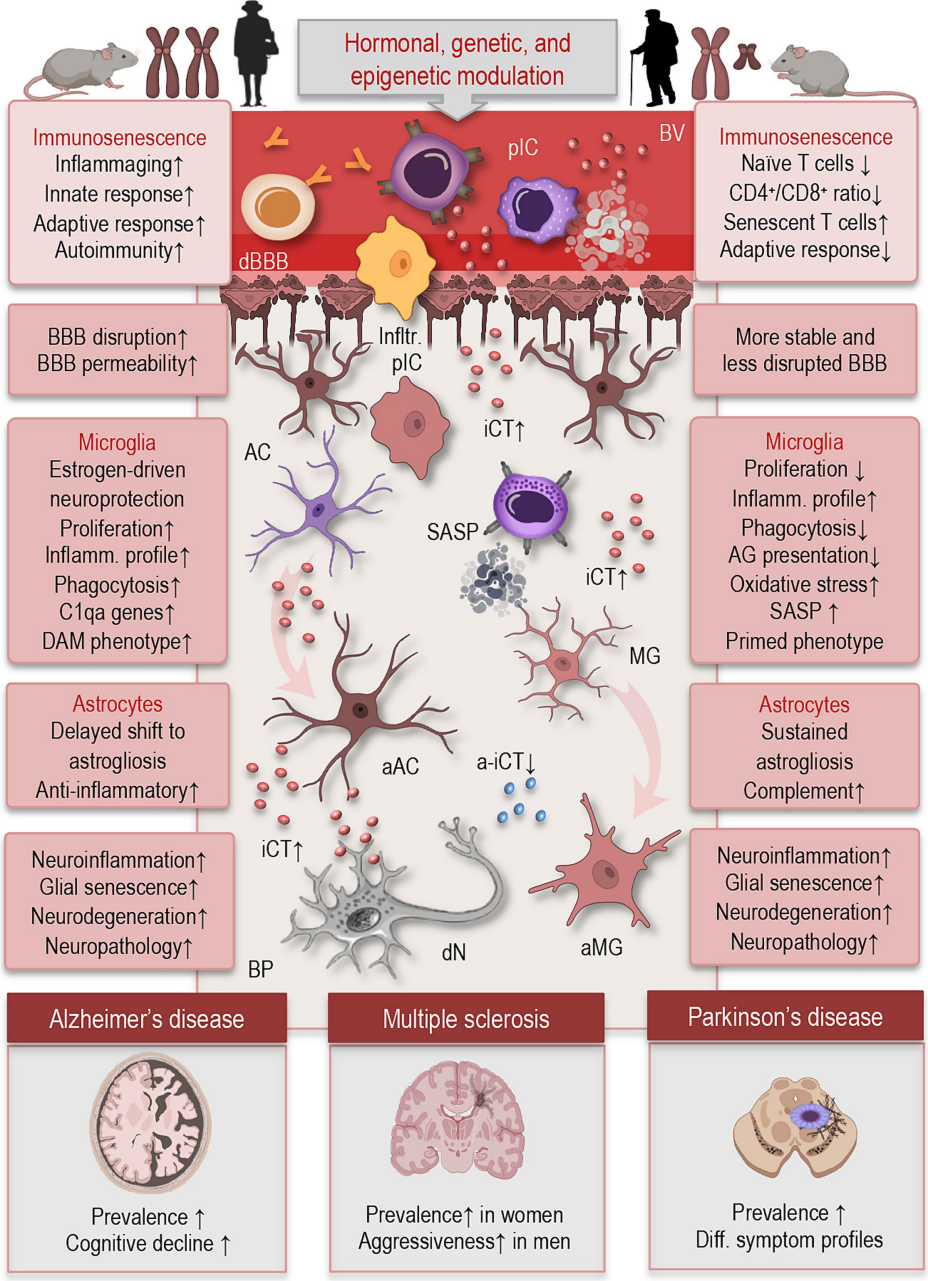
Simplified illustration of sex differences in immunosenescence and neuroimmune functions, based on data from human and rodent studies. Biological sex shapes immune and neuroimmune function through hormonal, genetic, and epigenetic mechanisms. Females (left) generally mount stronger innate and adaptive immune responses, with increased production of pro-inflammatory cytokines and higher susceptibility to autoimmune diseases. Aged men (right) exhibit a more rapid decline in naïve T cells, decreased CD4^+^/CD8^+^ ratios, and increased accumulation of senescent T cells, contributing to diminished adaptive immunity. In microglia, aging females exhibit delayed pro-inflammatory activation, higher expression of homeostatic markers, and enhanced phagocytic capacity, while aged males show earlier upregulation of inflammatory profile, and oxidative stress markers. Astrocytes in aged females retain greater anti-inflammatory signaling and delayed shift to astrogliosis, partially driven by estrogen, whereas aged male astrocytes display increased pro-inflammatory cytokine release, reduced neuroprotective functions, and enhanced complement activation. These sex-specific glial trajectories may impact vulnerability, progression, and symptom profiles in neuroinflammatory and neurodegenerative diseases such as multiple sclerosis, Alzheimer’s, and Parkinson’s disease. BV, blood vessel; pIC, peripheral immune cells; cBBB, compromised blood–brain-barrier; BP, brain parenchyma; Ifltr., infiltrating; iCT, inflammatory cytokines; a-iCT, anti-inflammatory cytokines; dN, degenerating neuron; MG, microglia; aMG, activated microglia; AC, astrocyte; aAC, activated astrocyte; SASP, senescence-associated secretory phonotype; DAM, disease-associated microglia; C1qa, complement system; CD, cluster differentiation.

Biological sex exerts a profound influence on both peripheral and central immune function, shaping responses to infection, injury, and age-related stressors. These differences emerge from the combined effects of sex chromosomes, gonadal hormones, and epigenetic regulation, and they persist across the lifespan (Figure 2). In the context of aging, sex-based differences in immune system dynamics and CNS surveillance contribute to divergent trajectories of neuroinflammation and susceptibility to neurodegenerative disease.

### 3.1 Sex-based differences in the peripheral and central immune systems

Women typically exhibit more robust peripheral immune responses than men, with higher baseline levels of circulating immunoglobulins, greater antibody responses to vaccines, and heightened T-cell activity (Di Benedetto et al., 2019; Klein and Flanagan, 2016). These enhanced responses are largely attributed to the immunostimulatory effects of estrogens and the presence of immune-related genes on the X chromosome. Conversely, men are more prone to infections and have a higher prevalence of some chronic inflammatory conditions, which may reflect lower immune reactivity but a greater tendency toward dysregulated inflammation (Giefing-Kroll et al., 2015).

At the level of the CNS, sex also shapes immune architecture and function. Microglia, the brain’s resident immune cells, differ morphologically and transcriptionally between sexes even under basal conditions. Studies in mice have shown that male microglia display a more pro-inflammatory phenotype in early life, while female microglia are more phagocytic and responsive to injury in adulthood (Villa et al., 2018). In humans, recent transcriptomic profiling has identified a subset of female-enriched and disease-associated microglia (FDAMic), which are more abundant in women with late-onset AD and correlate with disease severity (Wu et al., 2024). These cells exhibit signatures of activation and proliferation, MHC class II presentation, and amyloid-β binding, but appear functionally impaired in phagocytosis—likely due to compromised estrogen receptor signaling in an APOE4 genetic background. Thus, these baseline differences in microglial programming may have long-term implications for how each sex responds to neurodegenerative triggers during aging.

### 3.2 Immune system aging in males vs. females

The process of immune aging—or immunosenescence—manifests differently between sexes (Figure 2). In general, men exhibit a more rapid decline in naïve T cells, decreased CD4^+^/CD8^+^ ratios, and increased accumulation of senescent T cells, contributing to diminished adaptive immunity (Márquez et al., 2020; Di Benedetto et al., 2015). In contrast, women retain more effective immune surveillance for longer but also exhibit higher levels of chronic, low-grade inflammation—or inflammaging (Franceschi et al., 2018). These sex-specific patterns may help explain why women live longer on average but experience higher rates of certain autoimmune diseases and age-associated inflammatory conditions.

The hormonal shifts that accompany menopause and andropause further modulate immune aging. The decline in estrogens during menopause is associated with increased systemic inflammation and decreased neuroprotection, while reductions in testosterone in aging men have been linked to diminished immunomodulation and increased susceptibility to neuroinflammation (Gubbels Bupp and Jorgensen, 2018; Kissick et al., 2014; Bouman et al., 2004). Thus, sex hormones act not only during the reproductive years but continue to play a crucial role in shaping immune function and inflammatory trajectories across the lifespan, influencing how the brain responds to aging and disease-related stressors. Below, we begin by examining sex-specific differences in CNS immune surveillance and responses.

### 3.3 Sex-specific CNS immune surveillance and responses

Sexual dimorphism extends into CNS immune surveillance, where both microglia and astrocytes exhibit sex-dependent patterns of activation and reactivity (Figure 2). For example, aged female mice tend to show greater microglial proliferation and a more sustained inflammatory profile following immune challenge, whereas aged males often exhibit reduced microglial numbers but heightened baseline expression of pro-inflammatory cytokines. They display amplified responsiveness to ATP, evidenced by increased purinergic receptor expression and stronger baseline ionic currents. These findings suggest a sex-dependent potential for immune activation in the CNS (Guneykaya et al., 2018).

Glial reactivity may be shaped through hormonal influences, with estrogens exerting neuroprotective and anti-inflammatory effects—partly via NF-κB inhibition and IL-10 induction—while testosterone and its metabolites modulate glial activity through androgen and estrogen receptors, underscoring mechanistic differences that will be explored in more detail below (Villa et al., 2016; Giraud et al., 2010; Spence and Voskuhl, 2012).

In astrocytes, similar sex-specific responses have been observed. Aging female astrocytes may be more prone to enter reactive states associated with loss of neuroprotective functions and gain of pro-inflammatory signaling, contributing to heightened susceptibility to neurodegenerative diseases (Hanamsagar and Bilbo, 2016). Moreover, sex differences in BBB permeability and neurovascular unit integrity—such as greater BBB disruption and increased permeability observed in aging females—may enhance the infiltration of peripheral immune cells into the brain and amplify central neuroinflammatory responses compared to males (Weber and Clyne, 2021).

In addition to microglia and astrocytes, oligodendrocytes (OLs) and oligodendrocyte precursor cells (OPCs) also exhibit sex-specific differences relevant to CNS immune surveillance and repair. Female-derived OPCs in neonatal rodent models display greater proliferative capacity, migratory potential, and resistance to injury, whereas male OPCs show a stronger tendency toward differentiation and myelination (Yasuda et al., 2021). In demyelination models, females tend to exhibit more efficient remyelination and oligodendrocyte turnover compared to males, even in the absence of circulating gonadal hormones, indicating intrinsic sex-based differences in OL lineage dynamics (Li et al., 2006). Moreover, transcriptomic analyses of OPCs suggest sexually dimorphic expression of genes involved in stress response, metabolic regulation, and myelin remodeling (Yasuda et al., 2021). Hormonal modulation further influences these cells: estrogens and progesterone enhance OL survival and remyelination, while testosterone promotes myelin repair and attenuates neuroinflammation (Breton et al., 2021; Hussain et al., 2013). These findings underscore the importance of including OLs and OPCs in sex-informed analyses of CNS immunity, especially in demyelinating and age-associated neurodegenerative disorders.

Together, these findings further highlight the need for sex-aware research frameworks to better capture the full spectrum of glial responses and vulnerabilities associated with aging and neuroinflammatory conditions.

## 4 Hormonal regulation of neuroinflammation

Sex hormones—primarily estrogens, androgens, and progesterone—play a central role in shaping the brain’s immune landscape. Their effects extend beyond reproductive physiology to influence the function and phenotype of glial cells, BBB integrity, and inflammatory signaling cascades throughout the lifespan (Bonkhoff et al., 2025; Arevalo et al., 2015). These hormones modulate microglial surveillance, astrocytic support functions, and the expression of both pro-and anti-inflammatory mediators. For instance, estrogens have been shown to enhance anti-inflammatory signaling, while androgens and progesterone exert context-dependent effects on glial activity via their respective receptors (Zarate et al., 2017).

Changes in circulating hormone levels during menopause and andropause significantly modulate neuroinflammatory trajectories. The withdrawal of estrogens in women and the gradual decline of androgens in men alter glial cell reactivity, BBB integrity, and cytokine profiles. These hormonal shifts contribute to a heightened pro-inflammatory milieu in the aging brain, increasing susceptibility to neurodegenerative diseases such as AD, MS, and PD, often in a sex-specific manner (Cattaneo and Pagonabarraga, 2025; Gilli et al., 2020; Zarate et al., 2017; Vegeto et al., 2008).

To disentangle these complex interactions, we first consider the general, age-independent actions of estrogens, androgens, and progesterone on glial cells before turning to how age-related hormonal changes influence glial responses and neuroinflammatory outcomes in aging males and females.

### 4.1 Estrogens, androgens, and progesterone in glial modulation

Estrogens, particularly 17β-estradiol, have been widely documented to exert anti-inflammatory and neuroprotective effects in the central nervous system. They influence microglial activity by downregulating pro-inflammatory cytokine production (e.g., TNF-α, IL-1β) and upregulating anti-inflammatory factors like IL-10 and TGF-β. Estrogens also modulate astrocyte reactivity by inhibiting NF-κB signaling and promoting glutamate uptake, thereby preventing excitotoxicity and oxidative stress (Bonkhoff et al., 2025; Arevalo et al., 2015; Vegeto et al., 2008; Camon et al., 2024). These effects are mediated through estrogen receptors ERα, ERβ, and the G-protein-coupled estrogen receptor (GPER), which are differentially expressed in glial populations and modulate distinct transcriptional programs.

Androgens, such as testosterone and dihydrotestosterone, influence neuroimmune interactions in the brain through both androgen receptor (AR)-dependent and estrogen-mediated mechanisms. In male rodents, androgens have been shown to suppress microglial activation and reduce neuroinflammatory responses following injury or infection (Bonkhoff et al., 2025; Dengri et al., 2025). The immunosuppressive effects of testosterone may also involve conversion to estradiol by aromatase within the brain, suggesting an indirect role for estrogens even in males.

Progesterone, another key sex steroid, exhibits complex and context-dependent effects on glial function. It can suppress inflammatory responses in activated microglia and astrocytes by modulating Toll-like receptor signaling and inhibiting the release of pro-inflammatory mediators. Furthermore, progesterone has been implicated in promoting myelin repair and oligodendrocyte differentiation, indicating additional neuroprotective mechanisms relevant to aging and neurodegeneration (Fedotcheva et al., 2022; Zhou et al., 2024; Kolatorova et al., 2022).

### 4.2 Menopause, andropause, and neuroinflammatory trajectories

The decline in gonadal hormones that occurs during menopause in women and andropause in men alters systemic and central immune profiles. In postmenopausal women, a decline in estrogen levels has been linked to increased expression of pro-inflammatory cytokines such as TNF-α, IL-1β, and IL-6, which in turn diminishes microglial homeostasis and accelerates cognitive decline—particularly in AD models (Zarate et al., 2017; Zhang et al., 2024). Experimental studies further demonstrate that estrogen deprivation—through aging or ovariectomy—leads to heightened neuroinflammation, with significant increases in gliosis, cytokine expression in the hippocampus, and exaggerated immune responses to stimuli such as lipopolysaccharide (LPS) in aged females (Benedusi et al., 2012). Moreover, estrogen withdrawal impairs BBB integrity: tight junction proteins (e.g., claudin-5, ZO-1) are downregulated, and permeability increases, facilitating peripheral immune infiltration and contributing to glial senescence within the CNS (Maggioli et al., 2016). Together, these findings highlight how the postmenopausal loss of estrogen creates a pro-inflammatory state in the brain, disrupting neuroimmune balance and enhancing vulnerability to neuropathology.

In aging men, circulating testosterone gradually decreases, a trend that correlates with rising levels of systemic inflammatory markers such as IL-6, TNF-α, and C-reactive protein—reflecting an enhanced state of inflammaging (Maggio et al., 2005; Porcher et al., 2021). Emerging evidence suggests that lower testosterone compromises hippocampal integrity, with studies showing reduced synaptic plasticity and cognitive performance in conjunction with androgen decline (Pan et al., 2016). Concurrently, microglia in aging males exhibit a primed state—characterized by heightened sensitivity and exaggerated cytokine responses to stimuli—implying that reduced androgenic signaling may underlie an amplified neuroinflammatory profile, though these changes typically unfold more gradually compared to the sharper hormonal shifts seen in postmenopausal women (Norden and Godbout, 2013; Dengri et al., 2025).

Thus, age-related changes in sex hormone levels—particularly the decline in estrogens and androgens—profoundly shape the neuroimmune environment, modulating glial function, BBB integrity, and susceptibility to neuroinflammatory insults in a sex-specific manner. These hormonally driven differences, however, are further shaped by genetic and epigenetic mechanisms that program sex-specific immune and neural responses throughout the lifespan—topics we explore in the next section.

## 5 Genetic and epigenetic factors driving sex-specific neuroimmune responses

Sex-based disparities in neuroinflammation are influenced not only by hormonal fluctuations but also by inherent differences in genetic architecture and epigenetic regulation (Figure 2). These mechanisms shape immune and glial responses from early development through aging, contributing to sex-specific disease vulnerability.

### 5.1 X chromosome, gene dosage, and immune modulation

The X chromosome is significantly larger and more gene-rich than the Y chromosome and harbors numerous genes essential for immune function. Unlike most autosomes, females have two X chromosomes, and although one undergoes X-chromosome inactivation (XCI), several immune-related genes escape this silencing, resulting in dosage differences that can amplify immune responses in females (Lopez-Otin et al., 2023; Feng et al., 2024). This partial escape from XCI contributes to enhanced immune surveillance but also to increased vulnerability to immune overactivation and autoimmunity.

Key examples include TLR7 and TLR8, both pattern recognition receptors encoded on the X chromosome. While TLR7 is well-known for its role in viral RNA sensing and heightened expression in females, TLR8 similarly recognizes single-stranded RNA and has been shown to escape XCI in immune cells, leading to elevated expression and increased inflammatory potential in females (McCarthy et al., 2017; Feng et al., 2024). These receptors are expressed in microglia and other CNS-resident immune cells, where sex-dependent expression patterns may influence neuroinflammatory thresholds and disease susceptibility.

Another critical X-linked gene is FOXP3, a master regulator of regulatory T cells (Tregs), which modulate immune tolerance and prevent excessive inflammation. FOXP3 expression differences between sexes—potentially influenced by XCI skewing—may result in sex-specific regulatory immune capacity and could affect microglial reactivity and peripheral-CNS immune crosstalk during aging (Feng et al., 2024). Notably, Treg-derived IL-10 suppresses microglial activation and shifts their phenotype toward a neuroprotective state. For example, cerebral Treg cells attenuate LPS-induced microglial responses via IL-10-mediated downregulation of pro-inflammatory cytokines (Xie et al., 2015). In ischemic injury models, Tregs secrete osteopontin, promoting microglial transitions into protective and reparative states via integrin-β1 signaling, thereby facilitating tissue repair (Shi et al., 2021). Furthermore, in Alzheimer’s disease mouse models, peripheral Treg expansion via IL-2 treatment mitigates amyloid pathology and reduces glial reactivity by enhancing microglial phagocytic activity and fostering an anti-inflammatory environment (Faridar et al., 2022). Together, these studies underscore that Tregs—through IL-10 and osteopontin or other factors—play a critical role in maintaining microglial homeostasis and mediating peripheral-to-CNS immune interactions during aging and disease.

CXCR3, also located on the X chromosome, encodes a chemokine receptor involved in leukocyte trafficking and neuroinflammatory recruitment. It is expressed in both peripheral T cells and glial cells, and its dysregulation has been implicated in the pathogenesis of multiple sclerosis and Alzheimer’s disease. Studies have shown that CXCR3 expression is often higher in females, contributing to differential immune cell migration into the CNS and potentially influencing sex-specific disease courses (Feng et al., 2024; Oghumu et al., 2019).

Importantly, gene dosage—stemming from the differential number of X chromosomes between sexes—also influences neuroinflammatory and neurodegenerative outcomes. Studies show increased expression of X escapee genes (*Kdm5c*, *Kdm6a*) in aged female microglia, suggesting a dosage-dependent modulation of inflammatory signaling (Qi et al., 2021). Moreover, transcriptomic analyses reveal enrichment of X-encoded genes such as *Kdm6a*, *Eif2s3x*, and *Xist* in female hippocampal microglia with aging (Ocanas et al., 2023).

Experimental studies using the Four Core Genotypes (FCG) model, which separates sex chromosome complement (XX vs. XY) from gonadal sex, provide compelling evidence that two X chromosomes can exacerbate neuroinflammatory and neurodegenerative changes independently of sex hormones. In a 5xFAD Alzheimer’s disease model crossed with FCG mice, the presence of two X chromosomes (XX), regardless of gonadal type, was associated with heightened microglial activation, increased plaque burden, and neuritic dystrophy compared to XY genotypes (Casali et al., 2025). Additionally, separate studies in models of multiple sclerosis using EAE revealed that XY CNS complement mice displayed worse neuropathology—such as greater axonal and myelin loss—than XX counterparts, further supporting that sex chromosome complement can influence neuroimmune vulnerability (Voskuhl and Itoh, 2022). These findings substantively extend the concept of X-linked gene content by showing that X chromosome dosage—via differential complement—modulates microglial and neurodegenerative pathology independently of circulating hormone levels.

Together, these genes illustrate how the genomic architecture of the X chromosome contributes to sex differences in immune activation, glial function, and neuroinflammatory outcomes. Their differential expression—whether due to XCI escape, gene dosage effects, or hormonal regulation—shapes long-term immune programming and may underlie female-biased resilience or susceptibility in aging and disease.

### 5.2 Epigenetic regulation of neuroimmune function

Sex differences in epigenetic mechanisms—such as DNA methylation, histone modifications, and microRNA expression—play a central role in programming long-term immune and glial cell phenotypes and function. During early brain development, sex-specific DNA methylation patterns shape microglial and astrocyte programming, influencing inflammatory gene expression and cellular reactivity later in life. For instance, the demethylation of immune-related genes within the neonatal preoptic area in male rats—driven by lower DNA methyltransferase activity and androgen exposure—permits microglia-mediated inflammatory signaling, whereas elevated methylation in females suppresses this pathway, resulting in long-lasting differences in neuroimmune function (McCarthy et al., 2017; Chinn et al., 2021).

Global DNA methylation patterns in the aging brain undergo sex-dependent changes: females often maintain more stable methylation across genomic regions, while males exhibit greater variability—especially in immune and inflammatory gene loci—suggesting divergence in epigenetic control of neuroinflammation with age (Lopez-Otin et al., 2023; Shirokova et al., 2023; Yusipov et al., 2020).

Histone modifications—such as acetylation and methylation—are key epigenetic mechanisms that regulate chromatin accessibility and gene transcription (Bannister and Kouzarides, 2011). In the CNS, these modifications shape the transcriptional landscapes of glial cells, including microglia and astrocytes, thereby influencing their activation states and inflammatory responses. During aging and in neurodegenerative conditions, altered histone marks such as increased H3K27 acetylation or H3K4 methylation have been linked to persistent microglial priming and astrocytic reactivity. These chromatin-level changes contribute to exaggerated neuroinflammatory responses to stressors, and show evidence of sex-specific patterns, further highlighting the epigenetic complexity of neuroimmune regulation in aging brains. For instance, elevated H3K27 acetylation at inflammatory gene promoters in female microglia enhances transcriptional responsiveness to secondary insults, a process that appears less pronounced in males (Shirokova et al., 2023; Ramamurthy et al., 2022; Singh and Paramanik, 2024).

Transcriptomic analyses reveal that female hippocampal microglia undergo a more robust shift toward inflammation-associated pathways during aging than their male counterparts. Females show greater upregulation of complement genes (e.g., C1qa) and metabolic reprogramming toward glycolysis—hallmarks of the disease-associated microglial (DAM) phenotype—while aged males exhibit a more muted transcriptional response (Ramamurthy et al., 2022; Singh and Paramanik, 2024; Kang et al., 2024).

Recent findings have also highlighted sex-biased expression of epigenetic regulators, such as TET enzymes and DNA methyltransferases (DNMTs), particularly in aging glial cells. For instance, DNMT3A expression declines more significantly in aged male microglia, impairing silencing of pro-inflammatory genes and accelerating senescence-associated phenotypes (Chlamydas et al., 2022; Cisternas et al., 2020). In astrocytes, sex-linked histone acetylation patterns (e.g., H3K9ac) modulate inflammatory responses differently in male and female brains during aging and neurodegeneration (Giallongo et al., 2022).

Additionally, long non-coding RNAs (lncRNAs) and microRNAs (miRNAs) exhibit sex-specific expression profiles with age. miR-124 and miR-223, for instance, regulate microglial activation and show differential regulation in male vs. female brains, influencing the onset and progression of age-related neuroinflammation and diseases such as Alzheimer’s and multiple sclerosis (Qureshi and Mehler, 2012; He et al., 2023; Yang et al., 2023).

Collectively, these sex-specific epigenetic landscapes not only shape immune tolerance and plasticity but also influence susceptibility to chronic inflammation and neurodegenerative disease in later life. Understanding how these regulatory differences evolve with age provides essential insight into why men and women diverge in their neuroinflammatory profiles and disease trajectories.

## 6 Methodological considerations, research gaps, and future directions

Despite growing recognition of sex as a biological variable in neuroinflammatory research, substantial methodological limitations persist. A major concern is the historical underrepresentation of females in preclinical studies, particularly in rodent models of aging and neurodegeneration. For decades, male animals were predominantly used in neuroscience research due to perceived variability in female physiology related to hormonal cycles (Mamlouk et al., 2020; Will et al., 2017). A landmark review of eight biological fields—including neuroscience—found a male-to-female study bias of approximately 5.5 to 1 in rodents, with over 80% of studies excluding female subjects entirely—undermining our understanding of female neurobiology and inflammatory aging processes (Beery and Zucker, 2011). This bias has not only limited our understanding of sex-specific mechanisms but also skewed translational relevance, particularly in diseases like Alzheimer’s that disproportionately affect women (Beery and Zucker, 2011; Castro-Aldrete et al., 2023).

Modeling both aging and sex differences in animal studies presents complex challenges. Aging rodent models are resource-intensive, and replicating human-like hormonal transitions—such as menopause or andropause—is difficult. For example, rodents do not naturally undergo menopause, and ovary-removal models only partially mimic human sex steroid decline (Mamlouk et al., 2020; Will et al., 2017; Koebele and Bimonte-Nelson, 2016). Meanwhile, extending preclinical findings to humans is further complicated by species-dependent genomic and epigenomic differences in neuroimmune responses (Edler et al., 2021).

On the clinical side, sex-stratified analysis remains inadequate. Although guidelines like the NIH’s “Sex as a Biological Variable” policy have promoted inclusion, many trials still report combined results without separate analysis by sex (Waltz et al., 2021; Zhao et al., 2025). For example, recent drug trials in Alzheimer’s disease averaged results across both sexes, masking significant efficacy differences (e.g., 43% in men vs. 12% in women) (van Dyck et al., 2023). Therefore, a critical next step in clinical research is the consistent implementation of sex-stratified analyses in both observational and interventional studies.

Analyzing and interpreting complex biological systems—characterized by multifaceted, dynamic interactions across molecular, cellular, and systemic levels—remains a major challenge due to their high dimensionality, context dependence, and the nonlinear nature of their regulatory networks (Müller V. et al., 2019). Complex biological systems such as the immune system and CNS are composed of interdependent cellular and molecular components that interact across multiple organizational layers. Traditional single-layer network models, as applied in our previous work, revealed sex-and CMV-related differences in the topological properties of networks linking inflammatory biomarkers, hormones, neurotrophic factors, immune cells, and cognitive outcomes (Di Benedetto et al., 2019). However, these models are inherently limited in capturing inter-domain dependencies.

Multilayer network (MLN) models extend this framework by enabling simultaneous analysis of interactions within and across distinct layers—e.g., proteomic signaling, cellular communication, and tissue-level outcomes—thus offering a system-wide perspective of functional connectivity. In immune networks, nodes can represent immune cell types, with inter-layer links corresponding to signaling via cytokines, chemokines, or receptor–ligand pairs. Mass spectrometry and multiplex assays provide the data necessary to construct such networks, allowing inference of central hubs, signaling bottlenecks, and failure propagation paths (Müller et al., 2025). In the context of neuroinflammation, MLNs can uncover how perturbations in specific layers, such as glial reactivity or cytokine signaling, affect neuronal function and system resilience. Crucially, MLNs are well-suited to dissect sex-specific network architectures and dynamic responses, capturing how biological sex modulates connectivity strength, node centrality, and cascade vulnerability across layers. This approach holds promise for identifying sex-specific therapeutic targets and predicting network-level consequences of intervention strategies in age-related neuroinflammatory conditions.

Thus, only by systematically incorporating sex and age as core biological variables—rather than *post hoc* considerations—can research designs begin to reflect the complexity of neuroinflammatory processes in aging. This requires not only balanced representation of male and female subjects in both preclinical and clinical studies, but also analytical frameworks that allow for meaningful sex-and age-stratified interpretation of results. Integrating computational and systems-level methodologies, including but not limited to multilayer network models, offers a powerful avenue to disentangle the multifaceted biological interactions underlying neuroinflammation. These approaches can help to uncover hidden sex-dependent network structures, identify context-specific drivers of immune dysregulation, and predict differential treatment outcomes. Broader adoption of such methods will be critical to advancing precision medicine strategies that address sex-and age-related vulnerabilities, ultimately leading to more accurate, inclusive, and effective interventions for neurodegenerative diseases.

## 7 Conclusion

Understanding neuroinflammation in the aging brain requires a nuanced appreciation of sex as a fundamental biological variable. Accumulating evidence highlights distinct trajectories in how males and females experience immune aging, glial activation, and vulnerability to neurodegenerative processes. These differences arise from a complex interplay of genetic and epigenetic regulation, hormonal milieu, and environmental exposures—factors that shape not only the onset and progression of neuroinflammation but also the efficacy of therapeutic interventions.

The striking sex disparities observed in the prevalence, symptoms, and outcomes of conditions such as Alzheimer’s and Parkinson’s disease underscore the urgency of moving beyond a one-size-fits-all research paradigm. Despite increased awareness, critical gaps remain in both preclinical and clinical frameworks, particularly regarding sex-balanced study designs, age-appropriate models, and the reporting of sex-specific findings.

Addressing these challenges is not only a matter of scientific rigor but a prerequisite for advancing personalized, equitable approaches to the prevention and treatment of age-related neurodegenerative diseases. By fully integrating sex-based considerations into experimental design, analysis, and interpretation, the field can uncover more accurate disease mechanisms and develop targeted interventions that reflect the true biological diversity of the aging brain.
